# The Effect of Silicon Dioxide Nanoparticles Combined with Entomopathogenic Bacteria or Fungus on the Survival of Colorado Potato Beetle and Cabbage Beetles

**DOI:** 10.3390/nano12091558

**Published:** 2022-05-04

**Authors:** Elena I. Shatalova, Ekaterina V. Grizanova, Ivan M. Dubovskiy

**Affiliations:** 1 Siberian Federal Scientific Centre of Agro-BioTechnologies of the Russian Academy of Sciences, 630501 Krasnoobsk, Russia; 2 Laboratory of Biological Plant Protection and Biotechnology, Novosibirsk State Agrarian University, Dobrolubova Str. 160, 630039 Novosibirsk, Russia; katalasa_2006@yahoo.com; 3 Laboratory of Biotechnology of Microorganisms and Plants, Tomsk State University, 634050 Tomsk, Russia

**Keywords:** nanobiopesticide, biocontrol, plant protection, blastospore, *M. robertsii* metabolites, modified of silicon dioxide nanoparticles

## Abstract

Three types of modified silicon dioxide nanoparticles (SiO_2_, 10–20 nm) with additives of epoxy, silane and amino groups, used independently and in combination with the entomopathogenic bacteria *Bacillus thuringiensis* subsp. *morrisoni* and fungus *Metarhizium robertsii* were tested against Colorado potato beetle (*Leptinotarsa decemlineata*) and cabbage beetles (*Phyllotreta* spp.). All three nanoparticles were found to have an entomocidal effect on Colorado potato beetle larvae and crucifer flea beetles when ingested. Increased susceptibility of insects to *B.* *thuringiensis* or *M. robertsii* blastospores and their metabolites was shown after exposure to the modified silicon dioxide nanoparticles. The potential of modified silicon dioxide nanoparticles to enhance the efficiency of biopesticides based on the bacteria *B.* *thuringiensis* and fungi *M. robertsii* is considered in the paper.

## 1. Introduction

The unique properties and biological effects of nanomaterials (i.e., with diameters less than 100 nm) have become a popular topic for agricultural research in recent years [1]. Positive and negative effects provided by nanomaterials on plants have been described in detail [2,3]. Nanoparticles can be applied in the field to accelerate the destruction of pesticides in soil and water [4,5], and for crop protection by improving fertilizer efficiency [6]. The direct effects of silica nanoparticles (SiO_2_ NPs) on plant growth may be positive, non-significant or negative [7]. A positive effect of silicon nanoparticles on productivity and some physiological characteristics of plants has been noted: to overcome environmental stress on plants, proline concentration and the activity of the antioxidant enzymes were increased [7,8]. In two weeds *Amaranthus retroflexus* L. and *Taraxacum officinale* F. H. Wigg., as SiO_2_ NP treatment concentration increased, germination, root and shoot lengths, fresh and dry weights, and photosynthetic pigments as well as total protein decreased [9]. The known effects of SiO_2_ NPs on beneficial insects are limited. However, their effects on some associated natural enemies *Coccinella* spp., *Chrysoperla carnea*, and true spiders are negative, which might include the indirect effect of the poor quality of their prey that have been directly impacted by silica NPs [7,10]. Animal studies show a dual effect of silicon nanoparticles. The positive effect of SiO_2_ NPs supplementation of Nile tilapia (*Oreochromis niloticus* L.) via water resulted in a significant increase in growth and hematological parameters, as well as enhancement of antioxidant capacity (TAC), and an increase in immune related gene expression of IL-1β in the presence of SiO_2_ NPs [11]. Concurrently, the negative effect of SiO_2_ NPs on *O. niloticus* was shown through induced serum biochemical changes, histopathological alterations, and modulation of the gene transcription profile during long-term exposure [12]. The nanoencapsulation of pesticides is considered to increase their efficiency and environmental safety, and to improve the penetration of insecticides into pests [13]. Silicon, titanium dioxide, silver and zinc nanoparticles could be used in plant protection as nanopesticides for pest and disease control [14,15,16]. Nanoparticles of silicon, silver, aluminum, zinc and titanium were found to be effective at controlling the rice weevil *Sitophilus oryzae* and silkworm *Bombyx mori* [13,17].

Colorado potato beetle (*Leptinotarsa decemlineata* Say.) (CPB) and cabbage beetles (*Phyllotreta* spp.) are dangerous pests in Russia, Europe, North America and Africa [18,19]. CPB can propagate and acclimate in a wide range of habitats due to their high plasticity, migration capacity and intraspecific polymorphism [20].

Entomopathogenic bacteria *Bacillus thuringiensis* (*Bt*) and fungi *Metarhizium* spp., *Beauveria* spp., *Lecanicillium* spp. are widely used for pest control worldwide, and enhancing efficiency of these biological agents is important to reduce or replace the application of chemical non-eco-friendly insecticides [21]. Bt spores, Cry-toxins and other bacterial metabolites (vegetative and secreted insecticidal proteins, metalloproteases, chitinases, etc.) are used as active components for bioinsecticides [22]. For fungi *Metarhizium* spp., conidia, mycelium, and blastospores with metabolites can each be used for pest management [23]. The application of secondary metabolites as insecticides against the crop pests is promising because they are biodegradable, non-toxic to nontarget organisms and highly selective, and also have low resistance development in the target pest population [23]. The efficiency of bacteria *Bt* and fungi could be enhanced by using them as active components with nanopesticides to overcome the resistance and defense barriers of insects. SiO_2_ nanoparticles are able to reduce cuticle and intestine barriers, inhibit cell-mediated immunity and detoxification enzymes, and increase susceptibility to the product’s biological agents [24]. Tests have proved that nanosilicon synthesized by plants leads to activation of the *Bombyx mori* nuclear polyhedrosis virus (BmNPV) in silkworms [25]. *Bt* coated with ZnO nanoparticles hinders the development of larvae and pupae of the cowpea weevil *Callosobruchus maculatus* [26]. Bacteria *Bt* and fungi are employed to produce biogenic silver and gold nanoparticles against various insects including disease vectors [27,28,29,30,31].

This work aims to test three modifications of silicon dioxide nanoparticles (SiO_2_, 20–30 nm) with epoxy (1Si), silane (2Si) and amino (3Si) groups individually and in combination with bacteria *Bacillus thuringiensis* or fungi *Metarhizium robertsii* metabolites on the survival of Colorado potato beetle (*Leptinotarsa decemlineata* Say.) and cabbage beetles (*Phyllotreta* spp.).

## 2. Materials and Methods

### 2.1. Insect Collecting and Rearing

Experiments were carried out on two types of beetle (Order: Coleoptera): crucifer flea beetles (genus: *Phyllotreta* spp.) and the Colorado potato beetle (CPB) (*Leptinotarsa decemlineata*). Imagoes of crucifer flea beetles (*Phyllotreta* sp. L.) were collected from radish fields (*Raphanus sativus var. sativus*). The dominant species in the group were *Phyllotreta atra* L., subdominants were *Ph. undulata* Kutsch. and *Ph. vittula* Redt. The *Phyllotreta armoraciae* species were collected from the field with wild horseradish (*Armoracia rusticana*). The larvae of CPB were collected from potato fields free of insecticides in Moshkovsky District, Novosibirsk Region (Russia); the third instar larvae of CPB were used for experiments. Collected insects were maintained under laboratory conditions with 12/12 h light/dark cycle in plastic containers at 25 °C.

### 2.2. Bacteria and Fungi Cultivation

The fungus *Metarhizium robertsii* (strain 2017) (Mr) and bacterium *Bacillus thuringiensis* ssp. morrisoni var. thuringiensis (Btm19) (Bt) were used to infect insects by oral inoculation. Fungus Mr blastospores were produced by inoculating modified Czapek medium (per L: 20 g sucrose, 4 g peptone, 2 g NaNO_3_, 1 g KH_2_PO_4_, 0.5 g MgCO_4_ 7H_2_O; 0.5 g KCl, 10 mg FeSO_4_; pH 7.3 ± 0.2 at 25 °C) with conidia and incubating in a orbital shaker-incubator (130 rpm) for 3 days at 26 °C [32]. Fungal blastospores and their metabolites in culturing media were used for insect treatment Bacteria were cultured on plates of Luria-Bertani (LB) medium (1% trytone, 0.5% yeast extract, 1% NaCl in w/v, pH 7.0) at 30 °C until complete autolysis had occurred releasing the spores and the toxin crystals [33]. Spores and crystals of the bacteria were resuspended in 10 mM phosphate buffer (PBS) containing 150 mM NaCl, pH 7.2 and washed twice with saline solution (NaCl 0.9% w/v) at 6000× g for 10 min at 4 °C. Collected spore-crystal mixtures (1:1) were resuspended in PBS [34]. The titers of fungus and bacteria were counted in a hemocytometer.

### 2.3. Modifications of Silicon Dioxide Nanoparticles

Three types of modified silicon dioxide nanoparticles (SiO_2_, 10–20 nm) with epoxy (1 SiO_2_), silane (2 SiO_2_) and amide (3 SiO_2_) groups (10–30 nm) were tested. These had the following properties:

1: SiO_2_, 99.8%, surface modified with epoxy groups, dispersible, SiO_2_; particle size 10–20 nm; pH 6.0–7.5; surface area 90–130 m^2^/g. SiO_2_ nanoparticles containing epoxy groups can covalently interact with the primary amino, thiol, or hydroxyl groups of proteins. This reaction takes place in an alkaline medium (pH 9.0 and higher) due to the opening of the epoxy ring and does not require the addition of crosslinking agents. At acidic pH values, the epoxy ring may hydrolyze.

2: SiO_2_, 99%, treated with Silane Coupling Agents, SiO_2_. Nanopowder D50; particle size 10–20 nm; surface area SSA, >400 m^2^/g; silane content 1~2 wt %. In the case of SiO_2_ nanoparticles treated with Silane Coupling Agents, non-covalent bioconjugation of basic proteins and amino-containing fragments of DNA and RNA is possible [35].

3: SiO_2_, 99.8%, surface modified with amino groups, dispersible, SiO_2_ particle size 10–20 nm; pH 6.0–7.5; surface area 90–130 m^2^/g. The presence of amino groups on the surface of SiO_2_ nanoparticles provides the possibility of non-covalent bioconjugation of DNA and RNA fragments due to electrostatic interactions between amino groups in nanoparticles and phosphodiester internucleotide groups in nucleic acids.

The nanoparticles used in this work are commercially available from SkySpring Nanomaterials Inc., Houston, TX, USA (https://ssnano.com/; accessed on 1 April 2022) as Silicon Oxide Nanoparticles with catalogue number: epoxy (1 SiO_2_ #6852HN), silane (2 SiO_2_ #6811DL) and amide (3 SiO_2_ #6851HN) The antifungal and antibacterial activity of these nanoparticles against Mr and Bt has been tested and no antimicrobial activity was detected (see more information in Supplementary Materials Methods and Tables S1 and S2).

### 2.4. Experimental Design

Nanoparticles were tested as pure suspensions (1 mg per ml of phosphate buffer, (PBS) 10 mM, pH 7.2) and in combinations with bacteria (1 mg of nanoparticles per 1 mL of a suspension of Bt spore/crystals 10^6^ in PBS) or fungus (1 mg of nanoparticles per 1 mL of a suspension of fungus Mr blastospores 10^6^ in PBS). Nanoparticles combined with bacteria or fungi were sonicated for 1 min.

Oral inoculations of CPB larvae and crucifer flea beetle with nanoparticles, bacteria Bt, fungi Mr and their combinations were performed by a single dipping (10 s) of potato *Solanum tuberosum* and horseradish *Armoracia rusticana* leaves into PBS suspensions of nanoparticles, fungus, bacteria and their combinations. The control group of insects were fed with leaves treated with PBS.

The twenty larvae of CPB or fifteen imago of flea beetle were placed in glass jars or plastic containers (50 mL) with leaves of a feed plant (potato for CPB and horseradish for flea beetle) treated once with suspension of nanoparticles, bacteria Bt, fungi Mr or their combinations. The diet was replenished with a fresh leaf without treatment when appropriate. The data were recorded on the 1st, 3rd and 7th, 10th days. Sixty CPB larvae and forty-five flea beetle imagoes were tested for each treatment. The experiments were repeated three times independently.

### 2.5. Data Analysis

The data were analyzed using GraphPad Prism 8 (GraphPad Software Inc., San Diego, CA, USA). Survival was calculated using the product limit (Kaplan-Meier) method. Cox’s proportional hazards survival regression was used to quantify the differences in mortality rates.

## 3. Results

The study revealed that nanoparticles used independently and in combination with microorganisms had different effects on CPB larvae and crucifer flea beetles.

**Figure 1:**
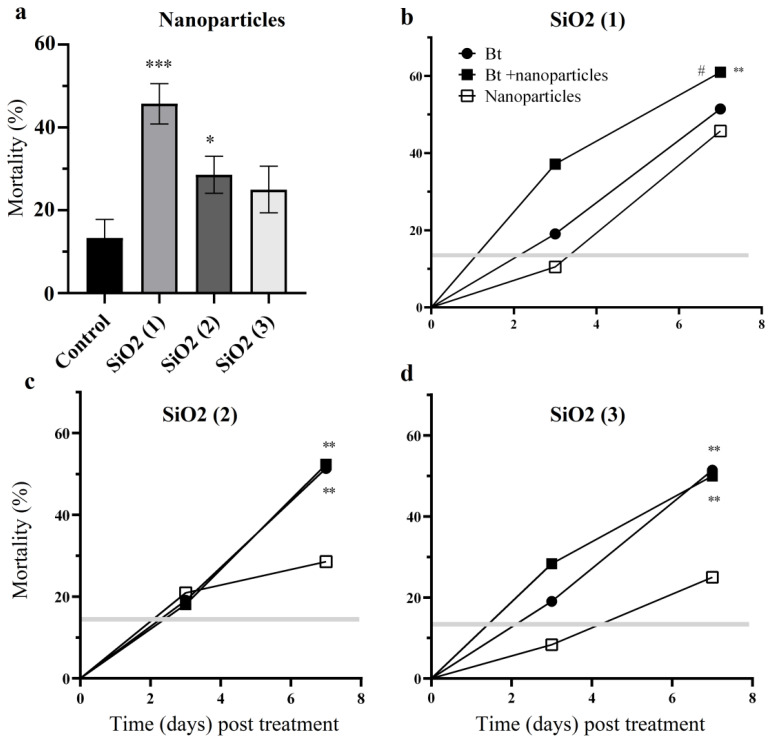
Survival of *Leptinotarsa decemlineata* larvae over seven days following treatment with silicon dioxide nanoparticles with epoxy SiO_2_ (1), silane SiO_2_ (2), amino SiO_2_ (3) groups (**a**), bacteria *Bacillus thuringiensis* (Bt) and combination (Bt+ nanoparticles) (**b**–**d**). The gray bar represents the mortality (mean) in control (* *p* < 0.05; ** *p* < 0.01, *** *p* < 0.001 compared with native larvae (Control) (**a**); (** *p* < 0.01 compared with treatment by nanoparticles, # *p* < 0.05 compared with treatment by Bt) (**b**–**d**).

**Figure 2:**
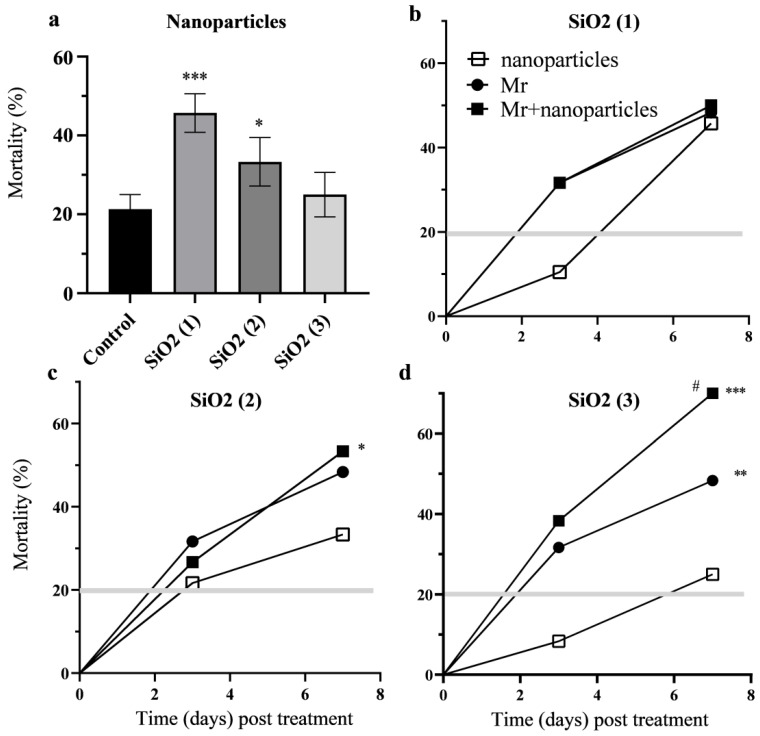
Survival of *Leptinotarsa decemlineata* larvae over seven days following treatment with silicon dioxide nanoparticles with epoxy (1 SiO_2_), silane (2 SiO_2_), amino (3 SiO_2_) groups (**a**), fungus *Metarhizium robertsii* (Mr) and combination (Mr + nanoparticles) (**b**–**d**); The gray bar represents the mortality (mean) in control (* *p* < 0.05; ** *p* < 0.01; *** *p* < 0.001 compared with treatment by nanoparticles, # *p* < 0.05 compare with treatment by Mr) (**b**–**d**).

Treatment of CPB larvae with SiO_2_ (1) and SiO_2_ (2) nanoparticles lead to a significant (*p* < 0.001, *p* < 0.05) increase in the mortality ~46% and ~30% respectively when compared with controls over the 7-day experimental period (Figure 1a and Figure 2a). Treatment of CPB larvae with the combination of Bt bacteria and SiO_2_ (1) nanoparticles resulted in a significant 10–15% increase in the mortality when compared with treatment with Bt (*p* < 0.05) and with nanoparticles (*p* < 0.01) (Figure 1b). It was shown that SiO_2_ (1) nanoparticles can accelerate Bt bacterial pathogenesis and cause the mortality rate to reach 37% on the 3rd day (Figure 1b). The accelerated infection process can significantly reduce damage to green parts of potato by CPB. The combination of Bt bacteria with SiO_2_ (2) nanoparticles caused no significant increase in the mortality of insects compared to Bt treatment (Figure 1c,d). The combination of the Mr blastospores, their metabolites and SiO_2_ (2) nanoparticles resulted in significantly (*p* < 0.05) higher mortality of CPB larvae compared with the treatment with nanoparticles (Figure 2c). Treatment of CPB larvae with the combination of the Mr blastospores, their metabolites and SiO_2_ (3) nanoparticles led to significantly higher mortality (*p* < 0.05) compared with the treatment with fungus and (*p* < 0.001) with nanoparticles (Figure 2d).

**Figure 3:**
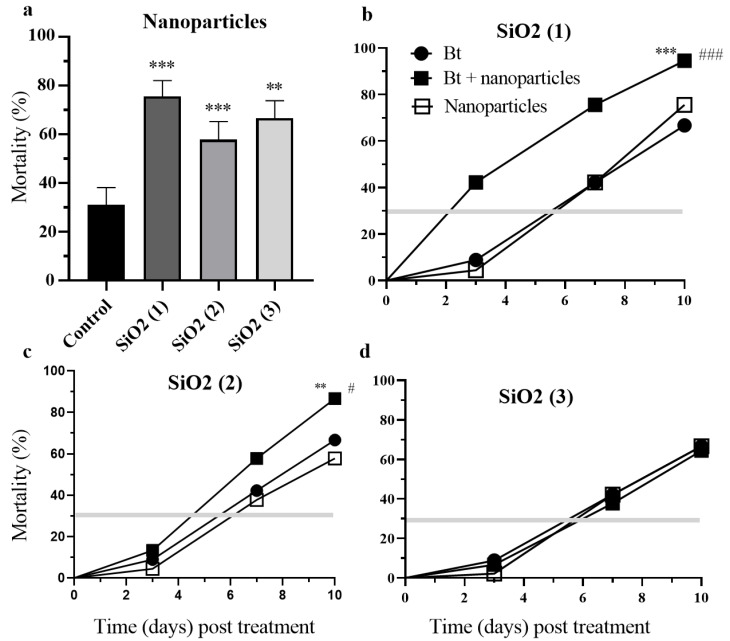
Survival of crucifer flea beetles *Phyllotreta* spp. imagoes over ten days following treatment with silicon dioxide nanoparticles with epoxy (1 SiO_2_), silane (2 SiO_2_), amino (3 SiO_2_) groups (**a**), bacteria *Bacillus thuringiensis* (Bt) and combination (Bt + nanoparticles) (**b**–**d**) (** *p* < 0.01, *** *p* < 0.001 compared with native beetles (Control) (**a**); (** *p* < 0.01 compared with treatment by nanoparticles, # *p* < 0.05 compared with treatment by Bt, ### *p* < 0.05 compare with treatment by Bt) (**b**–**d**).

**Figure 4:**
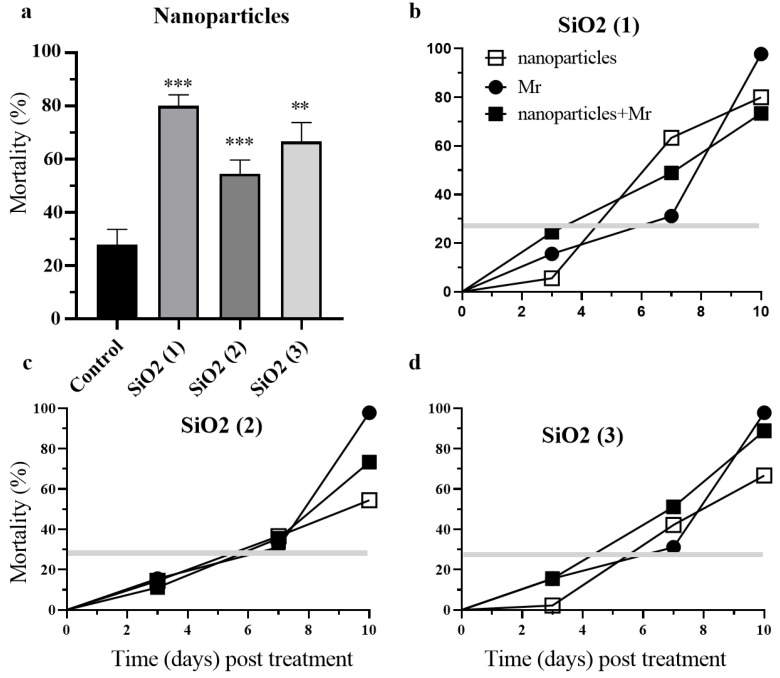
Survival of crucifer flea beetles *Phyllotreta* spp. imagoes over ten days following treatment with silicon dioxide nanoparticles with epoxy (1 SiO_2_), silane (2 SiO_2_), amino (3 SiO_2_) groups (**a**), fungus *Metarhizium robertsii* (Mr) and combination (Mr + nanoparticles) (**b**–**d**); ** *p* < 0.01; *** *p* < 0.001 compared with native beetles (Control) (**a**).

Treatments of crucifer flea beetles (genus: *Phyllotreta*) with SiO_2_ (1), SiO_2_ (2) and SiO_2_ (3) nanoparticles had significant (*p* < 0.001, *p* < 0.001,) negative and increased mortality up to ~75%, 60% and ~70% respectively as compared with control 25% over the 10-day experimental period (Figure 3a and Figure 4a). Within 10 days post treatments the combination of Bt bacteria and SiO_2_ (1) or SiO_2_ (2) nanoparticles significantly elevated the mortality rate of insects by 28% and 20% respectively as compared with treatment with Bt (*p* < 0.001, *p* < 0.01) and with nanoparticles (*p* < 0.01, *p* < 0.05) (Figure 3b,c). The insect mortality rate for the combination of Bt and SiO_2_ (2) nanoparticles had already reached 40% on the 3rd day post treatment (Figure 3b,c). The combination of the Mr blastospores, their metabolites and nanoparticles resulted in no significant difference in the survival of crucifer flea beetles during experiments (Figure 4b–d).

## 4. Discussion

The present study showed that the three types of modified silicon dioxide nanoparticles with epoxy (1 SiO_2_), silane (2 SiO_2_) and amide (3 SiO_2_) groups have an entomocidal effect on the CPB larvae and crucifer flea beetles when ingested. The mode of action and effect of nanoparticles against insects are dependent on the methods of application-ingestion or penetration of nanoparticles through the cuticle [36]. The impact of silica nanomaterials on insects is usually considered to be through their action through the cuticle. Nonetheless, for bumblebees *Bombus terrestris* L., exposure to silica nanoparticles resulted in midgut epithelial injury in affected workers [37] brought about by blocking the digestive tract and inducing malformation of external morphology [7]. The effectiveness of modified silicon dioxide nanoparticles against crucifer flea beetles is significant, making them promising for application in the field.

When combined, silicon dioxide nanoparticles modified with epoxy SiO_2_ (1), and silane groups SiO_2_ (2) with Bt bacteria lead to elevation in the mortality rate of insects. The combination of fungal blastospores and metabolites with silicon dioxide nanoparticles modified with amide group SiO_2_ (3) gave a raise of mortality rate of CPB larvae and crucifer flea beetles.

Increased susceptibility of insects to Bt or fungi blastospore and their metabolites when exposed to the modified silicon dioxide nanoparticles may be attributed to changes in intestinal permeability. Intestinal permeability, integrity and regeneration are some of key factors of insect susceptibility to Bt bacterial infection [38,39] It has been found that silicon nanoparticles increase the permeability of tissues for the active substance [40]. Some virulence factors of Bt demonstrate toxic effects in hemolymph after penetration through the intestinal tissue [41]. The permeability of the midgut is also important for mycosis because the blastospores ingested by the larvae are able infect the insect through the gut and rapidly invade the haemocoel [42]. In addition to the above, microorganisms and their metabolites become more adhesive to a leaf surface and insect setae, which leads to higher consumption of inoculum [43].

The impact of different nanoparticles on the antioxidant system of insects is clear when ingested, injected into haemocoel or applied topically [36]. It was demonstrated that titanium dioxide nanoparticles induced antioxidant and detoxification systems (malondialdehydes, glutathione S-transferases and superoxide dismutases) in *Galleria mellonella* larvae [44]. The effect of the combination of modified silicon dioxide nanoparticles and Bt could be based on the elevated level of oxidative stress in the larval midgut, -it is already well known that Cry toxins of Bt result in an antioxidant imbalance [45]. An imbalance in the antioxidant system could have an impact on the development of mycosis because secondary metabolites such as destruxins of *Metarhizium* directly and indirectly incapacitate the defense mechanism of insect hosts and accelerate the EPF infection process [46].

The properties of bio -pesticides can be improved as a result of nanoparticle effects on transport systems. Mobilization or combination of entomopathogens with nanoparticles could improve the penetration ability into gut tissue, adhesion in the gut, and possibly resistance to the enzymes and microbiota metabolites [47]. A similar effect with a reduction in microbial diversity of the intestine was shown for *Spodoptera litura* [48].

## 5. Conclusions

Our findings illustrated that modifications of silicon dioxide nanoparticles have an enhanced insecticidal effect on the Colorado potato beetle larvae and crucifer flea beetles when ingested in combination with *B. thuringiensis* bacterial spores and crystals or *M. robertsii* fungal blastospores and metabolites. Taken together, the mechanisms of action of modified silicon dioxide nanoparticles need further research; however, these materials have potentially promising applications to enhance the efficiency of biopesticides based on entomopathogenic bacteria and fungi.

## Data Availability

Not applicable.

## Supplementary Materials

The following supporting information can be downloaded at: https://www.mdpi.com/article/10.3390/nano12091558/s1, Table S1: Information about nanoparticles used in the study; Table S2: Nanoparticles antifungal and the antibacterial activity against *Metarhizium robertsii* (Mr) and *Bacillus thuringiensis* (Bt).
