# Supplementary material for: The Effect of Silicon Dioxide Nanoparticles Combined with Entomopathogenic Bacteria or Fungus on the Survival of Colorado Potato Beetle and Cabbage Beetles
Source: Nanomaterials (Basel). 2022 May 4;12(9):1558. doi: 10.3390/nano12091558 (PMC9100758; doi:10.3390/nano12091558)
Supplement: Supplementary file 1 [file nanomaterials-12-01558-s001.zip › nanomaterials-1689926-supplementary.pdf]

nanomaterials

MDPI

Supplementary Materials

The Effect of Silicon Dioxide Nanoparticles Combined with Entomopathogenic Bacteria or Fungus on the Survival of Colorado Potato Beetle and Cabbage Beetles

Elena I. Shatalova [1,2,*], Ekaterina V. Grizanova [2,3] and Ivan M. Dubovskiy [1,2,3,*]

1 Siberian Federal Scientific Centre of Agro-BioTechnologies of the Russian Academy of Sciences, 630501 Krasnoobsk, Russia
2 Laboratory of Biological Plant Protection and Biotechnology, Novosibirsk State Agrarian University, Dobrolubova Str. 160, 630039 Novosibirsk, Russia; katalasa_2006@yahoo.com
3 Laboratory of Biotechnology of Microorganisms and Plants, Tomsk State University, 634050 Tomsk, Russia
* Correspondence: elenashatalova@mail.ru (E.I.S.); dubovskiy2000@yahoo.com (I.M.D.); Tel.: +7-923-124-6896 (E.I.S)

Table S1. Properties of nanoparticles used in this study.

Product №	Modification	Product Properties	Images	Link
6851HN	Modified with amino group	Nanopowder, SiO_2, 99.8%, 10-20 nm<br>Loss on drying (%) (105 °C, 2h): <3<br>Loss on ignition (%) (950 °C, 2h): <10<br>SiO_2 content (dry basis) (%): >85<br>SiO2 content (950 °C, 2h) (%): >99.8<br>Carbon content (%): >0.3<br>Surface area (m²/g): 90-130<br>Bulk density (g/ml): 0.15	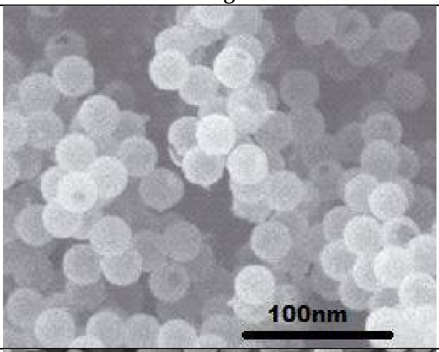	*3 SiO_2
6811DL	Treated with silane coupling agents (SiO_2, 99%,)	Nanopowder, SiO_2, 99%, 10-20nm<br><br>SiO_2 Nanopowder SSA: >400 m²/g<br>Silane content: 1~2wt%	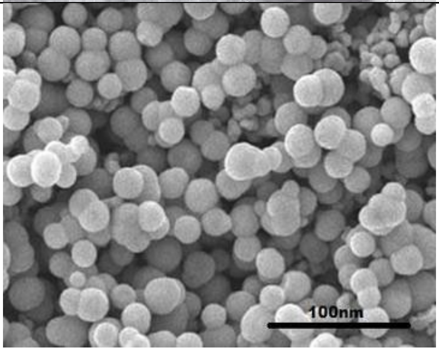	*2 SiO_2
6852HN	Modified with epoxy group	Nanopowder, SiO_2, 99.8%, 10-20 nm<br><br>Loss on drying (%) (105 °C, 2h): <3<br>Loss on ignition (%) (950 °C, 2h): <12<br>SiO_2 content (dry basis) (%): >85<br>SiO_2 content (950 °C, 2h) (%): >99.8<br>Carbon content (%): >0.3<br>Surface area (m²/g): 90-130<br>Bulk density (g/ml): 0.15	no picture	*1 SiO_2

Images were provided by supplier: *3 SiO_2. - https://ssnano.com/inc/sdetail/silicon-oxide-nanoparticles--nanopowder-modified-with-amino-group/363; *2 SiO_2 - https://ssnano.com/inc/sdetail/silicon_oxide_nanoparticles_99_0_/209; *1 SiO_2 - https://ssnano.com/inc/sdetail/silicon_oxide_nanoparticles/364.

Table S2. Nanoparticle antifungal and antibacterial activity against *Metarhizium robertsii (*Mr) and *Bacillus thuringiensis (*Bt). Data presented as the number of colony-forming units (CFU) per Petri dish, three replicates per treatment; Mean±SE, One -way ANOVA with Dunnett post-hoc comparison used for comparison of the nanoparticle effects on bacteria and fungi compared to the control (PBS treatment).

Product	Antibacterial (Bt) CFU; p value	Antifungal (Mr) CFU; p value
PBS (control)	1795 ± 84.13	2177 ± 107.4
1 SiO_2	1926 ± 18.41<br>$p = 0.3296$	2050 ± 50.33<br>$p = 0.4856$
2 SiO_2	1979 ± 69.29<br>$p = 0.1325$	2120 ± 70.00<br>$p = 0.8951$
3 SiO_2	1855 ± 38.75<br>$p = 0.8170$	2034 ± 33.88<br>$p = 0.4021$

S.1 Method for nanoparticle antimicrobial activity analysis

The antimicrobial activity of three modifications of silicon dioxide nanoparticles (with epoxy (1 SiO_2), silane (2 SiO_2) and amide (3 SiO_2) groups) was analysed against the fungus *Metarhizium robertsii* (Mr) and the bacterium *Bacillus thuringiensis ssp. morrisoni* var. thuringiensis (Bt). Fungus and bacteria (10^4 in PBS) were incubated with nanoparticles (1 mg per mL) for 4 h at 28 °C, and 250 µL cultured on plates of Luria-Bertani (LB) medium (for Bt) or Czapek medium (for Mr). CFU of bacteria and fungi were counted at 24 h and at 3 days respectively post cultivation at 28 °C.
